# Myocardial deformation in aortic valve stenosis: relation to left ventricular geometry

**DOI:** 10.1136/hrt.2009.172569

**Published:** 2009-08-25

**Authors:** Dana Cramariuc, Eva Gerdts, Einar Skulstad Davidsen, Leidulf Segadal, Knut Matre

**Affiliations:** 1 Institute of Medicine, University of Bergen, Bergen, Norway; 2 Department of Heart Disease, Haukeland University Hospital, Bergen, Norway; 3 Department of Surgical Sciences, University of Bergen, Bergen, Norway

## Abstract

**Objective:**

To assess left ventricular (LV) strain and displacement and their relations to LV geometry in patients with aortic stenosis (AS).

**Design:**

Cross-sectional echocardiographic study in patients with AS. Peak circumferential, radial and longitudinal strain, and radial, longitudinal and transverse displacement were measured by 2D speckle tracking. Severity of AS was assessed from energy loss index (ELI). LV hypertrophy was present if LV mass/height^2.7^ ≥46.7/49.2 g/m^2.7^ in women/men and concentric LV geometry if relative wall thickness ≥0.43. LV geometry was assessed from LV mass/height^2.7^ and relative wall thickness in combination.

**Setting:**

Department of Heart Disease, Haukeland University Hospital, Bergen, Norway.

**Patients:**

70 patients with AS (mean age 73±10 years, 54% women).

**Interventions:**

None.

**Main outcome measures:**

Association of regional and average LV myocardial strain and displacement with LV geometric pattern and degree of AS.

**Results:**

Average longitudinal strain was lower in the hypertrophy groups and correlated with higher LV mass index and relative wall thickness, lower stress-corrected mid-wall shortening and smaller ELI (all p<0.05). Average strain and displacement in other directions did not differ between geometric groups. In multivariate regression analysis, lower average longitudinal strain was associated with higher relative wall thickness (β=0.15), lower ejection fraction (β=−0.16), systolic blood pressure (β=−0.16) and energy loss index (β=−0.20) (all p<0.05) (R^2^=0.72). When relative wall thickness was replaced with LV mass, lower longitudinal strain was also associated with higher LV mass (β=0.21, p<0.05) (R^2^=0.73).

**Conclusions:**

In patients with AS, lower average longitudinal strain is related to higher LV mass, concentric geometry and more severe AS.

In patients with aortic stenosis (AS), it has been demonstrated that increased left ventricular (LV) load induces changes in LV geometry in order to allow preservation of a normal ejection fraction in spite of reduced long-axis excursion by M-mode echocardiography,^1^ and depressed LV myocardial systolic function.^2–4^ It is well known that LV geometry impacts both assessment of LV ejection fraction and myocardial mid-wall function.^4^ ^5^ However, less is known about the association between LV geometry and strain and displacement.

Strain and displacement can be derived from LV deformation analysis offering direct measurement of myocardial deformation (ie, thickening and thinning in the radial plane, and shortening and lengthening in the longitudinal and circumferential planes) and thus enabling assessment of regional or global myocardial function.^6–9^ Myocardial deformation can be assessed both by tissue Doppler imaging and 2D speckle tracking echocardiography,^10–12^. For this study we chose 2D speckle tracking which is angle-independent and allows faster post-processing and analysis of multiple segments simultaneously.^11^ ^13^

The aim of this study was to assess the impact of LV geometry on LV strain and displacement in patients with degenerative AS.

## Methods

### Study population

This study was prospectively planned for all patients with degenerative AS who had conventional and 2D speckle tracking echocardiography undertaken at the echocardiography laboratory, Haukeland University Hospital, Bergen, Norway as part of prospective clinical trial protocols in the time period April 2006–October 2007. A total of 70 patients were identified, and all agreed to participate in this study. Forty patients had asymptomatic AS and came for scheduled study echocardiograms in the Simvastatin Ezetimibe in Aortic Stenosis (SEAS) study at the Haukeland University Hospital study centre.^14^ Thirty patients had symptomatic AS and were recruited consecutively in the same period at the screening visit for a multicentre trial comparing the benefits of Mosaic Ultra versus Perimount Magna aortic supra-annular bioprostheses in AS.

Concomitant hypertension was defined as a history of hypertension reported by the attending doctor. Blood pressure was measured at the end of the echocardiographic examination by an arm-cuff sphygmomanometer.

All patients gave written informed consent to participate in the study, which was approved by the regional ethics committees.

### Conventional echocardiography

All examinations were performed using a Vivid 7 echocardiograph (GE Vingmed Ultrasound, Horten, Norway) equipped with a phased-array transducer following the previously published standard SEAS echocardiographic protocol.^15^

#### LV geometry

LV dimensions were measured in parasternal long-axis view according to the American Society of Echocardiography guidelines.^16^ LV mass was indexed for height^2.7^. LV hypertrophy was considered present if LV mass/height^2.7^ ≥46.7 g/m^2.7^ in women and ≥49.2 g/m^2.7^ in men, respectively. Relative wall thickness was calculated from posterior LV wall thickness/LV internal radius ratio at end-diastole and considered increased if ≥0.43.^17^ LV geometry was assessed from LV mass index and relative wall thickness in combination: patients with normal LV mass were divided into normal LV geometry and concentric remodelling groups, and patients with LV hypertrophy into eccentric and concentric hypertrophy groups, respectively.^18^

#### LV systolic function

LV endocardial systolic function was assessed by biplane Simpson's ejection fraction^16^ (low if <50%), and LV myocardial systolic function as stress-corrected mid-wall shortening (scMWS) calculated as the ratio of actual to predicted mid-wall shortening for the actual circumferential end-systolic stress.^19^ ScMWS was considered low if <87% in men and <90% in women.^20^

#### AS severity

Doppler assessment of AS included measurement of peak and mean transvalvular velocities and gradients; aortic valve area by the continuity equation, indexed for body surface area; and pressure recovery adjusted aortic valve area (ie, energy loss index (ELI)) calculated by a previously validated formula.^21^ Severe AS was defined as ELI ≤0.55 cm^2^/m^2^.^22^

### Speckle tracking echocardiography

Recordings of five consecutive heart cycles were used to analyse regional deformation on grey-scale images^12^ recorded from the parasternal LV short-axis (at the level of papillary muscles) and apical four-chamber views. The cardiac cycle with the best image quality and without any artefacts was selected for reporting results. The LV short-axis was divided into the anteroseptal, anterior, lateral, posterior, inferior and septal segments, and the LV apical four-chamber into basal, middle and apical segments in the septum and the lateral wall, respectively. The mean frame rate was 85±17 frames/s for parasternal short-axis views and 74±10 frames/s for apical views. Analyses were conducted using appropriate software (EchoPac version 6.1.0, GE Vingmed Ultrasound). The regions of interest were defined manually by marking the endocardial border and adjusting the region to include the whole LV wall thickness. The software then automatically detected the frame-to-frame motion of the natural ultrasound reflecting markers (speckles). The position of myocardial speckles followed the longitudinal, radial and circumferential direction of motion. Deformation was assessed by strain and displacement and calculated in all three directions: circumferential, radial and longitudinal strain (CS, RS, LS), as well as radial, longitudinal and transverse displacement (RD, LD, TD).^23^ Aortic valve closure was identified from the pulsed wave Doppler recording in the LV outflow tract. Results are reported as the peak during the whole cardiac cycle (peak strain and peak displacement). Strain measurements from the individual parasternal and apical segments were also averaged to obtain a global LV strain value.

### Statistical analysis

Data management and analysis were performed using SPSS 15.0 software. Data are presented as mean±SD for continuous variables and as percentages for categorical variables. The λ^2^ test was used to compare categorical variables and full-factorial two-way analysis of variance with Sidak's post hoc test to compare continuous variables, as appropriate. Univariate correlates of strain and displacement were identified by Pearson's correlation for normally distributed data. Predictors of higher peak strain were assessed in multiple linear regression analyses using an enter procedure with collinearity diagnostics. Results are presented as multiple R^2^ for the model and β coefficients for significant covariates. Two-tailed p<0.05 was considered significant both in univariate and multivariate analyses.

## Results

### Patient characteristics

The study population included 54% women, 46% hypertensive and 50% overweight patients (table 1). Clinical characteristics did not differ between the four LV geometric groups, even though there were trends for more men in the eccentric LV hypertrophy group and more hypertensive patients in the concentric LV hypertrophy group. Ejection fraction was normal in all but one patient, while scMWS was low in 79% of the population (table 2). The presence of concomitant coronary artery disease was identified by preoperative angiography in 17 of 30 patients with symptomatic AS, 16 of whom underwent subsequent combined coronary artery bypass grafting and aortic valve replacement.

**Table 1:** Clinical characteristics in the total study population and in the four groups of left ventricular (LV) geometric patterns

	All (n=70)	Normal LV geometry (n=19)	Concentric remodelling (n=14)	Eccentric hypertrophy (n=12)	Concentric hypertrophy (n=25)
Age (years)	73±10	68±12	74±10	72±8	75±8
Women (%)	54	63	57	33	56
Body mass index (kg/m^2^)	25.0±4.0	26.1±3.4	22.6±4.5	25.7±2.4	25.3±4.4
Overweight (%)	50	58	29	67	48
History of hypertension (%)	46	42	43	42	52
Systolic blood pressure (mm Hg)	150±21	147±20	153±20	149±27	150±20
Diastolic blood pressure (mm Hg)	80±12	83±10	81±8	80±15	78±12
Pulse pressure (mm Hg)	69±19	64±19	71±21	69±24	72±15
Heart rate (beats/ minute)	64±10	63±9	69±10	61±9	64±10

Data are mean±SD or percentage.

**Table 2:** Echocardiographic characteristics in the total study population and separately in the four groups of left ventricular (LV) geometric patterns

	All (n=70)	Normal LV geometry (n=19)	Concentric remodelling (n=14)	Eccentric hypertrophy (n=12)	Concentric hypertrophy (n=25)
LV end-diastolic diameter (cm)	4.53±0.73	4.71±0.57	4.02±0.43	5.24±0.71	4.33±0.69
Septum thickness (cm)	1.47±0.33	1.18±0.17	1.37±0.24	1.47±0.24	1.75±0.30
Posterior wall thickness (cm)	0.99±0.21	0.76±0.12	1.03±0.15	0.94±0.11	1.18±0.14
Ejection fraction (%)	64±6	63±6	67±6	63±9	64±5
ScMWS (%)	74.7±20.0	91.9±18.0*	70.1±15.9	77.4±15.2	62.8±16.6
Low scMWS (%)	79	42*	93	83	96
LV mass index (g/m^2.7^)	52±16	39±6	41±7	64±16	64±14
Relative wall thickness	0.46±0.15	0.32±0.05	0.51±0.07	0.36±0.05	0.57±0.17
Peak transaortic velocity (m/s)	3.88±0.91	3.19±0.58*	3.70±0.95	3.94±0.84	4.46±0.73
Mean transaortic velocity (m/s)	2.87±0.70	2.36±0.45*	2.70±0.71	2.93±0.63	3.34±0.60
Peak transaortic gradient (mm Hg)	63±30	42±15*	58±31	65±26	82±29
Mean transaortic gradient (mm Hg)	39±19	26±10*	35±19	40±16	51±19
Aortic valve area index (cm^2^/m^2^)	0.56±0.20	0.66±0.20†	0.57±0.19	0.59±0.22	0.46±0.15
ELI (cm^2^/m^2^)	0.64±0.25	0.79±0.27†	0.65±0.23	0.66±0.26	0.52±0.19
ELI ≤0.55	43%	16%‡	43%	42%	64%

Data are mean±SD or percentage.

Statistical significance is not reported for LV mass index and relative wall thickness as they are included in the definition of the four geometric patterns.

* p <0.001;

† p <0.01 and

‡ p <0.05 between groups.

LV, left ventricular; ELI, energy loss index; ScMWS, stress-corrected mid-wall shortening.

### AS severity

In the total study population, 30 (43%) had severe AS (mean ELI 0.44, range 0.40–0.47 cm^2^/m^2^). Comparing the LV geometric groups, there were significantly more patients with severe AS in the concentric LV hypertrophy group (table 2).

### LV geometry

LV hypertrophy was present in 53% of the patients: 17% with eccentric and 36% with concentric hypertrophy, respectively, and was slightly more prevalent in men: 59% than in 47% in women, NS. Normal LV geometry was found in 27% of patients, and concentric remodelling in 20%. Severity of AS increased and scMWS decreased progressively from the normal LV geometry group to the concentric remodelling group and further in the hypertrophy groups (all p<0.01). LV ejection fraction was low in one patient with eccentric LV hypertrophy who had an ejection fraction of 49% (table 2).

### Myocardial deformation in relation to LV geometry

From a total of 840 analysed segments, 12 parasternal segments and six apical segments were excluded owing to suboptimal myocardial tracking and poor image quality.

**Figure 1:**
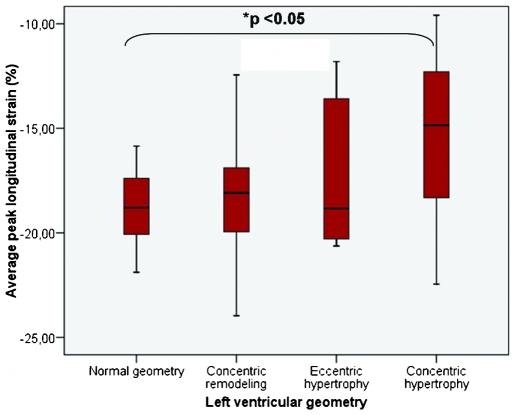
The range of average peak longitudinal strain in the four left ventricular (LV) geometric patterns. p<0.05 for comparison between four groups by full-factorial analysis of variance. *p<0.05 between average peak longitudinal strain in the concentric hypertrophy group versus the normal geometry group by Sidak's post hoc test.

**Figure 2:**
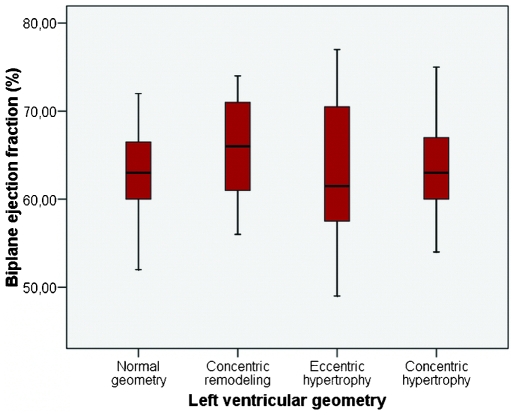
The range of ejection fraction in the four left ventricular (LV) geometric patterns. Comparison between four groups by full-factorial analysis of variance, and multiple comparisons by Sidak's post hoc test.

Average LS and TD differed significantly between the LV geometric groups. Average LS was lower in the hypertrophy groups, in particular in patients with concentric LV hypertrophy (figure 1), while average TD was highest in the eccentric LV hypertrophy group and lowest in patients with normal LV geometry (table 3). By contrast, no difference was found in LV ejection fraction between the geometric groups (figure 2). Average RS, as well as CS, tended to be lower in patients with eccentric hypertrophy (table 3). Average LS, but not CS or RS, was also lower in patients with obstructive coronary artery disease: −15±4% versus −17±3%, p<0.05.

**Table 3:** Average peak strain and displacement in the total study population and separately in the four groups of left ventricular (LV) geometric patterns

	All (n=70)	Normal LV geometry (n=19)	Concentric remodelling (n=14)	Eccentric hypertrophy (n=12)	Concentric hypertrophy (n=25)	p
Average LS (%)	−17±4	−18±3	−18±4	−17±3	−15±3*	0.05
Average CS (%)	−18±5	−18±5	−19±8	−17±3	−18±4	0.95
Average RS (%)	39±16	42±16	38±17	35±21	39±13	0.70
Average LD (%)	7±2	8±3	7±3	8±2	7±2	0.56
Average TD (%)	4±2	3±2	4±2	5±1‡	4±2	0.04
Average RD (%)	6±2	6±2	6±3	6±1	6±2	0.79

Data are mean±SD.

The p value for comparison between groups by two-way analysis of variance is shown in the last column.

* p<0.05 between average LS in the concentric hypertrophy group versus the normal geometry group, and between average TD in the eccentric hypertrophy group versus the normal geometry group by Sidak's post hoc test.

CS, peak circumferential strain; LD, peak longitudinal displacement; LS, peak longitudinal strain; RD, peak radial displacement; RS, peak radial strain; TD, peak transverse displacement.

**Figure 3:**
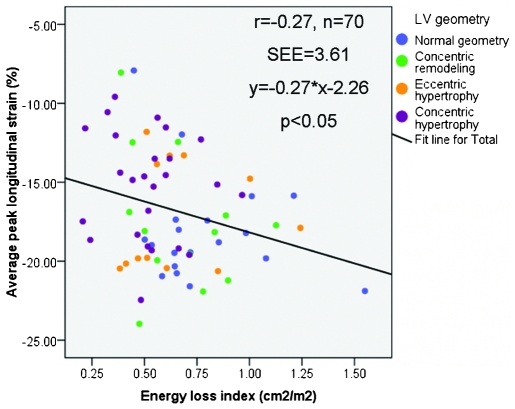
The correlation between average longitudinal strain and the severity of aortic stenosis by the energy loss index in the four left ventricular geometric groups. Pearson correlation coefficients r=−0.27, p<0.05.

In univariate analyses, lower average LS was associated with higher LV mass index (r=0.40) and relative wall thickness (r=0.33) (both p<0.01), and with smaller scMWS (r=−0.38, p<0.001) and ELI (r=−0.27, p<0.05, figure 3). The univariate correlation between LS and ejection fraction was r=−0.19, p=0.11. Lower average LD also correlated with higher relative wall thickness (r=−0.28, p<0.05). The other average strain and displacement parameters were not significantly related to LV geometry or the severity of AS.

In multiple regression analysis, lower average LS was associated with higher relative wall thickness, lower LV ejection fraction, systolic blood pressure and ELI, independent of gender, diastolic blood pressure or average LD (multiple R^2^=0.72, p<0.001) (table 4). When the model was run without LD among covariates, lower LS was still significantly associated with higher relative wall thickness and lower ELI (multiple R^2^=0.28, p=0.002). In a subsequent model, replacing relative wall thickness with LV mass, lower longitudinal strain was also associated with higher LV mass (β=0.21, p<0.05) (R^2^=0.73, p<0.001). Further, replacing average LD with scMWS among the covariates, lower scMWS remained the only significant covariate of lower average LS (β=−0.33, p=0.01) (R^2^=0.29, p<0.01). Age, presence of angiographic coronary artery disease, concomitant hypertension or mitral regurgitation were not significant covariates when added to these models.

**Table 4:** Predictors of average peak longitudinal strain (multiple R^2^=0.72, p<0.001)

Dependent variables	β	t	p
Relative wall thickness (%)	0.145	1.946	0.056
ELI (cm^2^/m^2^)	−0.204	−2.931	0.005
Ejection fraction (%)	−0.162	−2.295	0.025
Average peak LD (cm)	−0.697	−9.810	<0.001
Systolic blood pressure (mm Hg)	−0.161	−2.036	0.046
Diastolic blood pressure (mm Hg)	0.060	0.754	0.454
Male gender	0.199	2.826	0.006

ELI, energy loss index; .LD, peak longitudinal displacement.

### Regional myocardial deformation in AS

In the total study population, LS increased while LD decreased from base to apex both in the septum and in the lateral wall (both p<0.05, table 5). TD was lowest in the basal septum and highest in the basal lateral wall (table 5). Myocardial deformation varied regionally also in the circumferential and radial directions: CS was highest in the septum (−23±8%) and anteroseptum (−21±8%) and lowest in the lateral (−14±7%) and posterior walls (−14±8%), while RS and RD had the exact opposite variation: RS 42±19% and 44±20% in the lateral and posterior walls, and 34±16% in the anteroseptum; RD 7±3% both in the lateral and posterior walls, and 5±3% in the septum (all p<0.05). The segmental variation in LS, RS and CS was present both in patients with and without clinical evidence of obstructive coronary artery disease.

**Table 5:** Regional peak longitudinal strain and longitudinal and transverse displacement in the total study population

Septum	Basal	Mid-cavity	Apical
Peak LS (%)	−13±5*	−16±3	−22±7
LD (mm)	12±4*	9±4	4±2
TD (mm)	2±3‡	5±3	5±3
**Lateral wall**	**Basal**	**Mid-cavity**	**Apical**
Peak LS (%)	−14±6‡	−15±5	−20±8
LD (mm)	10±5*	6±4	2±3
TD (mm)	6±3†	4±2	4±2

Data are mean±SD.

* p <0.001

† p <0.01 and

‡ p <0.05 between basal, middle and apical segments.

LD, peak longitudinal displacement; LS, peak longitudinal strain; TD, peak transverse displacement.

**Figure 4:**
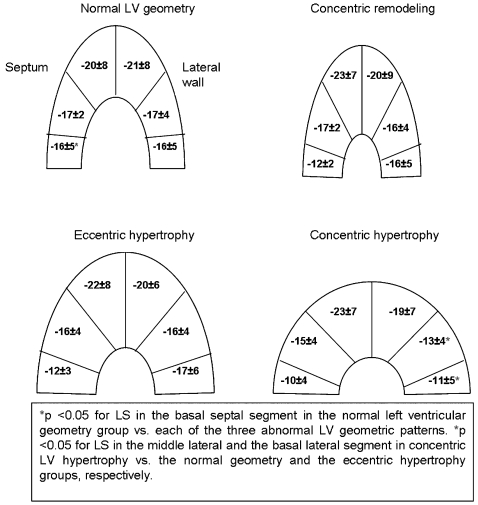
Peak longitudinal strain (LS) in the four left ventricular (LV) geometric patterns. Data are mean±SD. Comparisons are made by full-factorial analysis of variance with Sidak's post hoc test. p Values are corrected for multiple comparisons.

A comparison of the four LV geometric groups showed that LS was significantly lower in the basal septum in all abnormal LV geometric patterns compared with patients with normal geometry (figure 4). LS was also significantly lower in the middle and basal lateral wall in patients with concentric LV hypertrophy compared with the other LV geometric groups (figure 4). Furthermore, LD in the basal lateral wall was lower in the concentric groups (p<0.05). TD was significantly higher in basal septum in the eccentric hypertrophy group.

## Discussion

This study is the first to report multidirectional myocardial strain and displacement in relation to LV geometry in patients with degenerative AS. Our study has several interesting findings adding to current knowledge on the relation between LV geometry and systolic myocardial function in patients with AS. First, both LV hypertrophy and concentric geometry were independently associated with lower myocardial strain, in particular in the longitudinal direction, irrespective of the severity of AS. Second, lower average longitudinal deformation was associated with more severe AS. Finally, 2D speckle tracking analysis detected significant variations in regional strain and displacement between different LV segments.

In severe AS, changes in LV load induce LV geometric changes to sustain a normal ejection fraction despite decreased LV mid-wall mechanics.^2–4^ ^22^ Our study adds to previous findings by demonstrating that in patients with AS both increased LV mass and high relative wall thickness are associated with reduced myocardial longitudinal deformation in spite of normal global LV ejection fraction. In particular, in this study population, patients with concentric LV hypertrophy had the lowest average LS, demonstrating that this type of hypertrophy is characterised by low myocardial function whether measured by global myocardial longitudinal function or by scMWS. In chronic LV pressure overload by hypertension, our group and others have demonstrated that LV geometric patterns carry individual cardiovascular risk, independent of LV hypertrophy and well-known clinical risk factors, including increased body mass index, atrial fibrillation, diabetes or smoking.^24^ In contrast to findings in a post-myocardial infarction population, concentric LV geometry, in particular the concentric LV hypertrophy pattern, has been associated with depressed myocardial contractility as well as worse clinical outcome in hypertensive populations.^25^ ^26^ In AS, most publications on LV geometry have been based on studies in small groups of patients with severe, symptomatic AS, where up to 90% prevalence of concentric LV hypertrophy has been reported.^2^ ^27^ Previous publications have also suggested lower average LS to be a better marker of subclinical LV systolic dysfunction than LV ejection fraction or wall motion score index.^8^ ^28^ As demonstrated by our results, the association between average LS and LV mass was independent of gender, severity of AS and LV ejection fraction, and identified higher LV mass as a stronger covariate of lower LS than these variables. Our findings may help explain why concentric LV hypertrophy has been associated with higher in-hospital mortality after aortic valve replacement.^29^

Increasing severity of AS was, as expected,^30^ associated with higher prevalence of LV hypertrophy and more concentric geometry (table 2). However, in multivariate analyses, lower average LS remained significantly associated with more severe AS independent of LV geometry, demonstrating that reduced myocardial strain is not merely a reflection of LV geometrical changes in more advanced AS. Similar findings were previously reported in a small study of 32 patients with severe AS.^31^ In this study Bauer *et al* distinguished between LV hypertrophy with low peak systolic strain and LV hypertrophy with normal strain by tissue Doppler imaging, the first one being associated with higher risk of postoperative morbidity, while the second was considered benign compensatory hypertrophy.

As previously reported,^28^ average peak strain was independently related to LV ejection fraction, the most commonly used parameter of LV endocardial systolic function. LV ejection fraction could be regarded as rather a consequence than a predictor of longitudinal strain. However, the analysis of its relationship with LS is of practical importance since today's guidelines include LV ejection fraction in management decisions in patients with asymptomatic AS.

Average LV ejection fraction was, however, normal in this population and did not differ significantly between the four geometric groups, while both regional and average myocardial strain varied with LV geometry and degree of valve obstruction, probably reflecting more subtle, subclinical changes in LV myocardial function. Moreover, scMWS, which was a strong covariate of LS in our population, and LV longitudinal shortening, but not ejection fraction, have been previously shown to be correlated with symptoms in more advanced AS.^3^ ^32^ Increased mass with normal relative wall thickness (ie, eccentric LV hypertrophy) was in our study mainly associated with increased regional and average displacement in the transverse direction, but not with clinical evidence of obstructive coronary artery disease, possibly explaining the normal values of LV ejection fraction despite decreased myocardial strain.

Regional variation in myocardial deformation has been less studied in patients with AS. In our study, regional differences between the six LV segments in the longitudinal direction were detected in the total study population: increase in LS from base to apex accompanied by decrease in LD. The base-to-apex gradient in LS has been previously described in normal subjects, as well as in athletes and in patients with hypertrophic cardiomyopathy.^33^ However, segmental LS values were lower in our study than in findings in the 17 controls and 27 athletes recruited by Richand *et al*. Average LS was also much lower than that reported by Gjesdal *et al*^8^ in normal subjects, but well above the 14% threshold value suggested for identification of myocardial infarct in ischaemic cardiomyopathy.^8^ In a recent report by Lafitte *et al*,^34^ global LS was lower in patients with severe AS than in controls, and in particular associated with lower exercise capacity. Of note, the strain values reported in their paper are similar to those demonstrated in our study population. Moreover, in the study by Lafitte *et al*, a difference in average LS, but not CS or RS, was found between patients with AS and controls, which is in accordance with our results, and confirms previous data from tissue Doppler studies,^35^ as well as an earlier observation by Dumesnil *et al* on the dichotomy of the contraction components.^36^

In addition, we identified a variation in peak strain in the radial and circumferential directions: higher circumferential shortening and lower radial thickening in the septum and anteroseptum with opposite findings in the lateral and posterior walls (lower CS with higher RS). These results need further confirmation in future studies to determine if they are related to the AS itself or to other factors like LV structure or the position of the heart.

In conclusion, in patients with AS, changes in LV geometry, including increased LV mass and higher relative wall thickness, are associated with reduced LV regional and global myocardial deformation assessed by 2D speckle tracking, in particular in the longitudinal direction. Longitudinal peak myocardial strain is also progressively reduced with increasing severity of AS. LS is furthermore significantly related to previously validated and prognostically important measures of myocardial systolic function, such as scMWS. Our findings suggest that 2D speckle tracking may be used in clinical assessment of LV function in patients with AS to detect subclinical LV myocardial dysfunction which cannot be diagnosed by conventional echocardiographic measures of systolic function like LV ejection fraction. However, the prognostic implications of our findings remain to be assessed in future longitudinal studies.

## Study limitations

The definition of LV geometry was based on conventional parasternal measures of LV dimensions and wall thicknesses assuming the site of measurements to be representative for the whole ventricle. In spite of its limitations, this method is in accordance with current LV quantification guidelines, and has proven prognostic value in hypertensive patients.^24^

Myocardial strain and displacement are load-dependent measures of LV function. However, all patients included in the present analyses were asymptomatic and in stable haemodynamic condition at rest. 2D speckle tracking is dependent on image quality and high frame rate which were both high in the present study population as only 2% of the LV segments had to be excluded from analysis, and the average frame rate was >80 frames/s.^8^ ^9^

The thirty symptomatic AS patients recruited at the screening visit of the Mosaic Ultra versus Perimount Magna study underwent additional coronary angiography. However, the 40 patients recruited from the local SEAS study did not undergo coronary angiography or stress echocardiography as part of the protocol. We can therefore not exclude that some patients in this latter group had subclinical coronary artery disease. However, they did not present symptoms of obstructive coronary disease.

Antihypertensive treatment was not standardised in the study, but left to the discretion of the cardiac surgeons and general practitioners managing the individual patients. Thus, impact of antihypertensive treatment on deformation parameters could not be assessed in this study.

The present cross-sectional study could not evaluate whether LV ejection fraction, scMWS or longitudinal strain is the better marker of subclinical myocardial dysfunction. This important clinical question needs to be addressed in future prospective studies.
